# Gastrointestinal Cancer Found in the Cervix With Unknown Primary Site and Treated With Definitive Chemoradiation

**DOI:** 10.7759/cureus.27300

**Published:** 2022-07-26

**Authors:** Jasen Albana, Uma Goyal

**Affiliations:** 1 College of Medicine, University of Arizona, Tucson, USA; 2 Radiation Oncology, Banner MD Anderson Cancer Center, Phoenix, USA

**Keywords:** brachytherapy, radiation, chemotherapy, cervix, gastrointestinal cancer

## Abstract

Locally advanced gastrointestinal cancers and cervical cancers are usually treated with a multimodality approach. Our case report shows a patient who was found to have gastrointestinal cancer in the cervix, and no primary gastrointestinal cancer was found on workup. She underwent chemoradiation to the pelvis with concurrent capecitabine and then underwent cervical brachytherapy with tandem and ovoid. She initially had done well but then noticed increased symptoms at follow-ups. Unfortunately, she was found to have residual disease about 16 months after the completion of treatment. There have been no reports of treatment of gastrointestinal cancer in the cervix with an unknown primary site in the literature to our knowledge.

## Introduction

Cancer of unknown primary site (CUP) is defined as a group of metastatic tumors for which a standardized diagnostic workup fails to identify the site of origin at the time of diagnosis [1]. Approximately, 2%-5% of all cancer cases are CUP and 1% of the CUP cases are from the gastrointestinal (GI) tract. Early diagnosis of primary GI tract cancers is typically done through endoscopic examinations followed by a surgical biopsy of the area [2].

GI tract cancers are commonly found in areas such as the stomach, pancreas, esophagus, liver, and colon [3]. Therefore, it is atypical to find GI tract cancer in the cervix of a patient. There are two distinct types of cervical cancer: squamous cell carcinoma (SCC) and adenocarcinoma (AC). SCC is the most common kind of cervical cancer, encompassing almost 70% of all cervical cancers, and most occurrences of SCC are due to human papillomavirus infection (HPV). In contrast, adenocarcinoma comprises 20%-25% of all cervical cancers and is not as strongly related to HPV [4]. Pap smears are the conventional method for screening for cervical SCC, which is an effective and noninvasive method to test for any abnormalities in the epithelium of the cervix. Unfortunately, a pap smear test is not an effective method to screen for AC because AC is typically deeper in the cervix and requires a more invasive procedure. In addition, due to the lower rates of occurrence and case report documentation in AC (~10% of case reports involving cervical cancer), knowledge on AC’s natural history as well as optimized treatment is finite [5].

Thus, both cervical SCC and AC cancers undergo similar treatments. The standard treatment approach for locally advanced stages of these cancers is concurrent chemoradiotherapy (CCRT). Locally advanced cervical cancer (LACC), with or without lymph nodes, is characterized as a tumor within the cervix that is >4 cm in diameter that has not metastasized to other parts of the body (can range from stage IB2 to stage IVA). Another treatment is brachytherapy, in which a radioactive source is placed directly into or next to the tumor to deliver a high dose of radiation locally. However, SCC and AC do behave differently, and though the treatment plan is similar, the pattern of failures and recovery differs. Despite these differences, specific treatment strategies tailored to AC have not yet emerged [6]. One study found a significant difference in the survival probability between SCC and AC across a seven-year timeline in advanced stages [6]. The survival rate is also another factor that varies between AC and SCC. When the tumor size is <2 cm, the survival difference may be negligible; however, as the tumor size in AC increases, five-year survival rates then decrease in comparison to SCC (73% for SCC and 59% for AC) [5].

At our institution, we had a case of a patient found to have a histological GI adenocarcinoma in the cervix. Workup did not reveal the GI primary so it was treated similarly to a locally advanced cervical AC. Therefore, we provide a case study on a patient diagnosed with a histological GI adenocarcinoma found in the cervix who was treated similarly to a locally advanced cervical cancer. There have been no reports of treatment of GI cancer in the cervix with an unknown primary site in the literature to our knowledge.

## Case presentation

A 54-year-old Hispanic woman reported having diarrhea and increased number of bowel movements for the past four years. The patient began to follow up with her primary care physician in December 2019. The patient had previous surgical history including abdominoplasty in 2006, C section, and bilateral tubal ligation in 2000. In January 2020, pap smear results showed an atypical adenocarcinoma with an unknown origin. On February 5, 2020, the patient had endocervical curettage, cervical, and endometrial biopsies done to confirm atypical glandular cells. Pathology and staining showed that the adenocarcinoma was to be from the GI tract (negative for P16, vimentin, cytokeratin 20 (CK20), progesterone receptor (PR); positive for P53, CDX2, CK19). In order to find GI primary cancer, on February 25, 2020, MRI pelvis was done which showed a mildly T2 hyperintense endocervical mass measuring up to 2.5 cm extending into the upper ⅓ of the vagina. There were no suspicious pelvic nodes seen on imaging. The patient had a repeat biopsy showing focally positive PAX8, strongly positive CK7 and carcinoembryonic antigen (CEA), and weakly positive estrogen receptor (ER). Her tumor markers were within normal limits for CEA and carbohydrate antigen 19-9 (CA19-9). The patient also had a colonoscopy in March 2020 and an upper endoscopy, which showed no malignant tumor but did show *Helicobacter​*​​​​​​* pylori* in the stomach.

Her case was discussed at our multidisciplinary tumor board, where it was decided to treat the patient with GI oncology based on pathological findings. She then met with radiation oncology to discuss treating her similarly to a localized GI cancer versus locally advanced cervical cancer. Treatment discussion with the patient was that for cervical cancers, surgery may be recommended which may include a hysterectomy. However, given the GI origin of her cancer, surgery may not be recommended based on gynecology oncology discussion at tumor board. Given her good performance status and since no GI primary was found, it was decided to treat with definitive chemoradiotherapy including high-dose rate (HDR) brachytherapy, similar to locally advanced cervical cancer, to deliver a higher dose to the cervical location. After meeting with GI medical oncology, it was decided to give concurrent capecitabine.

**Figure 1:**
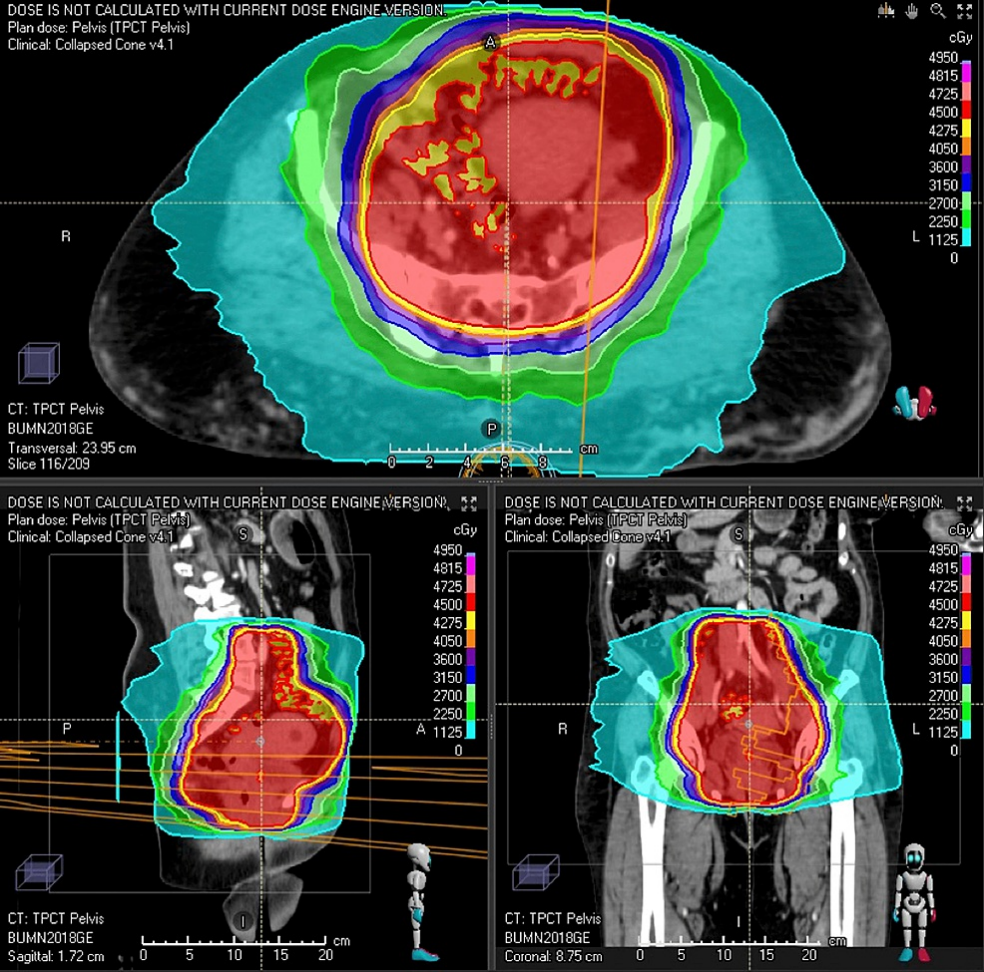
Radiation treatment plan External beam radiation treatment plan of the pelvis including lymph nodes and cervix/uterus/vagina which went to 45 Gy in 25 fractions. This plan was followed by brachytherapy to the cervix. TPCT: treatment planning computed tomography; cGy: centigray; A: anterior; P: posterior; R: right; L: left; S: superior; I: inferior.

In April 2020, the treatment delivered was 45 Gy in 25 fractions (fx) radiotherapy to the pelvis as shown in Figure 1. Concurrent capecitabine 1,500 mg twice daily on the days of radiation was prescribed. Then in June 2020, the patient received 5.5 Gy x 5 fx in biweekly HDR brachytherapy with tandem and ovoid procedures. She completed treatment in approximately eight weeks total. The patient tolerated the treatments well and was scheduled for a one-month follow-up.

**Figure 2:**
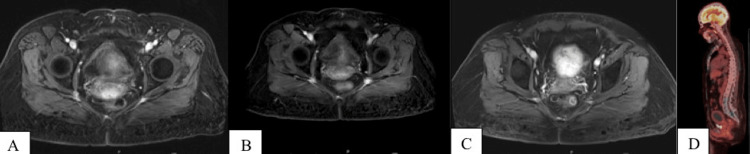
Imaging pre- and postchemoradiation (A) MRI pelvis June 3, 2020: axial T1 with contrast. (B) MRI pelvis September 23, 2020: axial T1 with contrast. (C) MRI pelvis September 22, 2021: axial T1 with contrast. (D) December 3, 2021 positron emission tomography (PET)/CT: sagittal view with avidity in vagina and cervix.

In September 2020, MRI pelvis results found a slight decrease in mass size from 2.5 cm to 2.1 cm and the patient also reported feeling “super”. Figures 2A-2D show imaging prior to chemoradiation and during follow-ups. At further follow-ups, the patient noted increased vaginal discharge and she underwent endometrial and cervical biopsies in October 2021 due to cervical stenosis showing adenocarcinoma of GI primary (negative PAX8 and positive CDX2).

In December 2021, she underwent a positron emission tomography (PET)/CT showing hypermetabolic foci at the cervix concerning for residual neoplasm and cervical stenosis with fluid in the endometrial cavity. There was also an ill-defined avidity at the vaginal canal. Her case was discussed at multidisciplinary tumor board and recommended for further chemotherapy using bevacizumab, capecitabine, and oxaliplatin versus surgery. The recommendations were discussed with the patient, and she refused chemotherapy and also felt that the surgery recommendation was too extreme for her regarding removing other organs. She requested a second opinion for treatment recommendations but had not met with another physician as of March 2022.

## Discussion

This is the first case study to our knowledge that looks at a histological GI tract cancer in the cervix that was treated similarly to a locally advanced cervical cancer for radiation therapy. GI cancers are primarily adenocarcinoma histologically and tend to be treated with a 5-fluorouracil (5FU)-based chemotherapy regimen and external beam radiation. However, given that our patient was found to have GI origin adenocarcinoma in the cervix, we treated her as a locally advanced cervical AC for radiation therapy, but she received concurrent capecitabine used in GI cancers for systemic therapy. Given that locally advanced cervical cancer radiation treatments generally include HDR brachytherapy and GI cancer treatments do not, it was thought that brachytherapy would provide a higher dose locally to the cervix which may increase our patient’s local control chances. Initially, she tolerated treatment well but unfortunately was found to have residual disease about 16 months after the completion of chemoradiation. Although there have been overall improvements in treatment outcomes for patients presenting at a locally advanced stage, approximately 50% experience recurrence within the first two years [7].

As shown in Table 1, there have been few published studies regarding treatment for cervical cancer in the GI tract and vice versa [8-9]. In our case study, no primary GI cancer was able to be found through imaging and endoscopic workup. Imachi et al. showed that metastatic gastric cancer to the cervix was found to have a poor prognosis despite treatment method [8]. Sassi et al. provided a rare case of metastatic colon cancer to the cervix, and surgical treatment was recommended [9]. In our case, since surgery was not recommended, we proceeded with definitive chemoradiation and brachytherapy, which showed residual disease about 16 months after the completion of treatment.

**Table 1:** Unpublished and published reports of gastrointestinal cancer metastasized to gynecological sites and vice versa

Author	Synopsis	Conclusions
Abstract: Qureshi T, Shobassy M, Pappas S. Cervical Cancer Presenting in an Extremely Unexpected Location; October 2017	Female patient with anorexia and weight loss for several months. Upon endoscopic biopsy, findings were consistent with metastatic squamous cell carcinoma originating from cervix to the duodenum. The patient offered treatment of palliative chemotherapy but declined and returned to Honduras for treatment.	The patient’s immunostains were positive for P16 and P40, while negative for cytokeratin 7 (CK7), PAX8, and CDX2. Findings were consistent with metastatic squamous cell carcinoma originating from cervix.
Imachi et al. 1993 [8]	Ages ranged from 29 to 57 years, and 81.3% of the patients were premenopausal. All patients received a colposcopy exam, which showed the presence of tumor cells determined to be metastatic adenocarcinoma. The prognosis was poor regardless of the treatment method.	Upon colposcopy exams, 57.1% were normal, but 56.3% had abnormal cervical smears. All patients in the study showed tumor cells present in the cervix.
Sassi et al. 2021 [9]	Thirty-eight-year-old North African Caucasian woman treated for a nonmetastatic colon adenocarcinoma. The patient had sigmoidectomy and incomplete adjuvant chemotherapy. Six months later, she showed vaginal bleeding caused by a cervical tumor. The patient then underwent decompressive and hemostatic radiotherapy.	Uterine cervix metastasis from primary colon adenocarcinoma is rare. Resection remains the standard protocol for the local treatment of resectable metastatic disease.
Current case report: Albana and Goyal 2022	Fifty-four-year-old Hispanic female who was found to have gastrointestinal cancer in the cervix and no primary gastrointestinal cancer was found on workup. She underwent chemoradiation with capecitabine per colorectal recommendations and then underwent cervical brachytherapy with tandem and ovoid per cervical recommendations.	She initially had done well but then noticed increased vaginal discharge at follow-ups. Unfortunately, she was found to have residual disease about 16 months after the completion of treatment.

## Conclusions

Our case study shows GI origin cancer in the cervix with unknown GI primary that was treated definitively with chemoradiation and brachytherapy. Due to the rarity of this diagnosis, optimal chemotherapy and radiation regimens for this case remain unknown. Further studies on treatment options for metastatic disease to the cervix based on primary cancer histology may be warranted.

## References

[1] Losa F, Soler G, Casado A (2018). SEOM clinical guideline on unknown primary cancer (2017). Clin Transl Oncol.

[2] Varadhachary GR (2007). Carcinoma of unknown primary origin. Gastrointest Cancer Res.

[3] Vedeld HM, Andresen K, Eilertsen IA (2015). The novel colorectal cancer biomarkers CDO1, ZSCAN18 and ZNF331 are frequently methylated across gastrointestinal cancers. Int J Cancer.

[4] Hu K, Wang W, Liu X, Meng Q, Zhang F (2018). Comparison of treatment outcomes between squamous cell carcinoma and adenocarcinoma of cervix after definitive radiotherapy or concurrent chemoradiotherapy. Radiat Oncol.

[5] Gien LT, Beauchemin MC, Thomas G (2010). Adenocarcinoma: a unique cervical cancer. Gynecol Oncol.

[6] Zhang S, Xu H, Zhang L, Qiao Y (2020). Cervical cancer: epidemiology, risk factors and screening. Chin J Cancer Res.

[7] Bandyopadhyay A, Mukherjee U, Ghosh S, Ghosh S, Sarkar SK (2018). Pattern of failure with locally advanced cervical cancer: a retrospective audit and analysis of contributory factors. Asian Pac J Cancer Prev.

[8] Imachi M, Tsukamoto N, Amagase H, Shigematsu T, Amada S, Nakano H (1993). Metastatic adenocarcinoma to the uterine cervix from gastric cancer. a clinicopathologic analysis of 16 cases. Cancer.

[9] Sassi I, Ghalleb M, Chemlali M, Mbarek M, Charfi L, Chargui R, Rahal K (2021). Uterine cervix metastasis from primary colon adenocarcinoma: a case report and review of the literature. J Med Case Rep.

